# Adiposity measures, lean body mass, physical activity and mortality: NHANES 1999–2004

**DOI:** 10.1186/1471-2369-15-108

**Published:** 2014-07-08

**Authors:** Sankar D Navaneethan, John P Kirwan, Susana Arrigain, Jesse D Schold

**Affiliations:** 1 Department of Nephrology and Hypertension, Glickman Urological and Kidney Institute, Cleveland Clinic, 9500 Euclid Avenue- Q7, Cleveland, OH 44195, USA; 2 Cleveland Clinic Lerner College of Medicine of CWRU, Cleveland Clinic, Cleveland, OH, USA; 3 Department of Pathobiology, Lerner Research Institute, Cleveland Clinic, Cleveland, OH, USA; 4 Department of Quantitative Health Sciences, Cleveland Clinic, Cleveland, OH, USA

**Keywords:** Obesity, Physical activity, Muscle mass, Kidney disease and death

## Abstract

**Background:**

Obesity and physical inactivity are major public health problems. We studied the associations between measures of adiposity, lean body mass, leisure time physical activity (LTPA), and death in those with and without chronic kidney disease (CKD).

**Methods:**

Associations between body mass index (BMI), waist circumference (WC), percent body fat, lean body mass (assessed with Dual-Energy X-ray Absorptiometry[DEXA]), leisure time physical activity (LTPA) and death were examined using the National Health and Nutrition Examination Surveys (NHANES 1999–2004). All-cause mortality was ascertained by linkage of NHANES files with the National Death Index.

**Results:**

9,433 non-CKD participants and 2,153 CKD participants who had fat mass measured using DEXA, BMI, WC, LTPA and mortality data were included. After adjusting for demographics, comorbid conditions, kidney function measures, C-Reactive Protein (CRP), and sodium intake there was no significant risk for death noted with higher WC, fat mass and BMI in those with and without CKD. When examining normal, overweight, and obese groups based on BMI criteria, being overweight (BMI 25–29.9 kg/m^2^) was associated with lower risk of death in those without CKD (Hazard ratio 0.62, 95% CI 0.40, 0.95). Higher lean body mass was associated with lower risk for death in those without kidney disease but not in the CKD population. There was a significantly higher risk for death among those who did not meet the minimum LTPA goals compared to those who met or exceeded the recommended activity levels (>450 MET/min/week) in those with and without CKD (CKD Hazard ratio: 1.36, 95% CI 1.003, 1.85; non-CKD HR 1.65, 95% CI 1.21, 2.26).

**Conclusions:**

In a representative sample of the US population, higher LTPA levels and lean body mass were associated with lower mortality in those without kidney disease. In CKD, higher LTPA was associated with lower risk of death. There was no association between adiposity measures and death in those with and without CKD except for lower mortality associated with overweight among those without CKD. The data suggests the need to develop programs to facilitate an increase in physical activity in people with and without kidney disease.

## Background

Obesity rates have increased in the past few decades along with an increase in comorbid disease states related to obesity and health care expenditures [1]. In the general population, being overweight (BMI 25–29.9 kg/m^2^) and class I obesity (BMI 30–34.9 kg/m^2^) are associated with lower mortality [2]. Some studies have also suggested that measures of abdominal adiposity such as waist circumference (WC) and waist-to-hip ratio might be better predictors of adverse outcomes in the general population [3,4]. Higher BMI is associated with an increased risk for development and progression of kidney disease [5,6]. However, reports indicate that higher BMI is associated with lower mortality in those with established CKD (reverse epidemiology) [7,8]. Recent population based studies have shown that WC rather than BMI might be a better measure for predicting mortality in those with CKD [9]. This could be attributed to the inability of BMI to distinguish muscle mass and fat mass as differential associations between muscle mass, fat mass and mortality might exist. However, studies examining the relationship between body fat, lean body mass (measured using dual-energy x-ray absorptiometry [DEXA], CT scan or other modalities) and mortality in those with CKD are lacking.

Several studies in the general population reported a lower risk for death among individuals with higher levels of physical activity [10-13]. Analyses using NHANES III data (1988–1994) suggested that inactive or insufficient physical activity levels are associated with lower death among those with and without kidney disease [14,15]. However, these analysis lacked fat mass and fat free mass data and whether higher physical activity levels have similar benefits despite accounting for fat mass and lean body mass is unknown. We hypothesized that obesity as measured by - higher percent fat mass, lower lean body mass and lower leisure time physical activity (LTPA), are associated with increased mortality in those with and without CKD. Therefore, we studied the associations between adiposity measures (BMI, WC and percent body fat), lean body mass and LTPA with all-cause mortality among a nationally representative sample of US adults.

## Methods

### Study population

**Figure 1:**
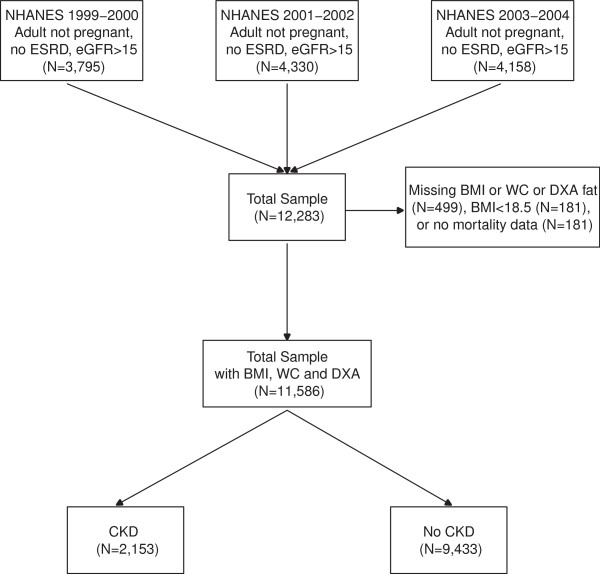
Flow chart of how patients were selected for the analysis.

We examined data from the National Health and Nutrition Examination Survey (NHANES), a nationally representative, complex and multistage probability survey of the US civilian, non-institutionalized population conducted by the National Center for Health Statistics. The National Centers for Health Statistics Ethics Review Board approved the study protocol and each participant provided written informed consent. Participants in NHANES were interviewed in their homes and underwent a standardized physical examination in a mobile examination center. Self-reported information on demographics, socioeconomic status, health conditions, health behaviors and routine site of healthcare were obtained during the interview. The examination component consisted of medical, dental, and physiological measurements, as well as laboratory tests administered by highly trained medical personnel. The three, 2-year cycles of the continuous NHANES 1999–2000, 2001–2002, and 2003–2004 were combined for this analysis. 11,586 participants who met the following criteria were included: 20 years of age and older who underwent medical examination, those who were not pregnant, had BMI and WC measured, had body composition measured or imputed using DEXA results, had a BMI ≥18.5 kg/m^2^, had serum creatinine and albumin/creatinine ratio results, and those who were not on dialysis and had eGFR ≥15 ml/min/1.73 m^2^ (Figure 1).

### Measures

#### Kidney disease and comorbidities

Participants without CKD and those with stage 1–4 CKD were included (Stage 1–2 CKD: those with urinary albumin-to-creatinine ratio of ≥30–299 mg/g [microalbuminuria] and ≥300 mg/g [macroalbuminuria]) with estimated glomerular filtration rate [eGFR] ≥60 ml/min/1.73 m^2^, or stage 3–4 CKD: eGFR 15–59 ml/min/1.73 m^2^). eGFR was calculated according to the Chronic Kidney Disease Epidemiology Collaboration (CKD-EPI) equations, using calibrated creatinine [16]. Serum creatinine levels were corrected for the 1999–2000 survey as suggested in the NHANES serum creatinine documentation [17]. Urine albumin-to-creatinine ratio (UACR) was calculated from spot urine albumin and creatinine samples. Diabetes was defined as self-reported if ever told by a doctor that the participant had “diabetes or borderline diabetes”. Hypercholesterolemia was defined as the presence of total cholesterol >200 mg/dl and/or use of cholesterol lowering medications. Liver disease was defined by the answer “yes” to the question, “Have you ever been told that you had any liver condition” Hypertension was defined as systolic blood pressure >140 mm Hg or diastolic blood pressure >90 mm Hg, or use of antihypertensive medications. Cancer was defined as self-reported by the question “ever told you had cancer or malignancy.”

#### Adiposity measures

Trained health technicians used standardized techniques and equipment to measure height and weight during the health examination conducted in each survey period. Waist circumference was measured midway between the lowest rib and the iliac crest with the participant standing. We classified male participants with WC >102 cm and female participants with WC >88 cm as obese [18]. BMI was calculated as weight in kilograms divided by the measured height in meters squared and we classified participants as normal if they had BMI 18.5-24.9 kg/m^2^, overweight if they had BMI 25–29.9 kg/m^2^, and obese if they had a BMI ≥30 kg/m^2^.

Body fat and lean body mass were measured using DEXA in NHANES 1999–2004 surveys. DEXA scans were administered to eligible subjects as part of the medical examination. Females who were pregnant did not receive the test. The whole body DEXA scan was acquired using a Hologic QDR 4500A fan-beam densitometer (Hologic, Inc., Bedford, Massachusetts) following the manufacturer’s acquisition procedures. Hologic DOS software version 8.26:a3* was used to acquire all scans; scanning was done in the fast mode. Because missing and invalid DEXA data were not missing at random, NHANES analysts performed multiple imputation to fill in missing or invalid DEXA data and the NHANES website provides 5 multiply imputed datasets for DEXA analysis [19]. Based on the WHO definition, male participants with >25% body fat and females with >35% body fat were classified as obese.

#### Lean body mass

Lean body mass excluding bone mineral content data was obtained from DEXA performed in the NHANES surveys.

#### Physical activity

Detailed information about leisure time physical activity (LTPA) over the past 30 days was obtained during the interview at home. Each LTPA was classified to a MET score according to the Compendium of Physical Activities [20]. LTPA was categorized into less than minimum goal (<450 MET/min/week), meeting the minimum goal (450 to <750 MET/min/week) and exceeding the recommended goal (>750 MET/min/week) [21].

#### Mortality data

NHANES Linked Mortality public use files are available for continuous NHANES periods 1999–2004. The follow-up time is from survey participation until December 31, 2006. Mortality status is based on a probabilistic match between NHANES and NDI death certificate records.

#### Statistical analysis

Demographic characteristics, body measurements and comorbidities between participants with and without CKD were compared using t-tests for continuous variables and Rao-Scott chi-square tests for categorical variables. Cox proportional hazards analyses were used to assess the association between different body measurements (BMI, WC, percent fat and lean body mass) and mortality while adjusting for age, gender, race, smoking status, income, salt intake, log c-reactive protein, cancer, eGFR and log albumin/creatinine ratio. Covariates were selected based on previous studies in this area and their clinical relevance. We assessed adiposity measures as continuous variables and also classified into categories as described previously [22]. We tested 2-way interactions between each of the continuous body measurements and age, gender and race in the adjusted models as these associations might differ based on these factors. Obesity contributes to the development of diabetes and hypertension which are known risk factors for CKD, cardiovascular disease and death. Since diabetes and hypertension are in the causal pathway of associations between obesity and death, these variables were not adjusted for in the multivariate models. We evaluated whether adiposity measures might be associated with mortality in different ways for those with and without each of those comorbidities by testing 2-way interaction terms in separate models. We also ran Cox models with the previously mentioned factors (except salt intake) plus LTPA below the minimum recommended level, and tested 2-way interactions between LTPA and each of the continuous adiposity measures. 2-way interactions between physical activity and age, gender and race in the model adjusted for percent fat were tested. We also evaluated whether LTPA might be associated with mortality in different ways for those with and without diabetes and hypertension by testing 2-way interaction terms in separate models.

All analyses were performed using survey procedures with SAS version 9.3 for Unix (SAS Institute, Cary, North Carolina), which account for the sampling design of NHANES and appropriately weight participants in statistical models. Diet weights were used for analyses including sodium intake while medical examination weights were used for all others. All analyses including DEXA results were run separately on each of the 5 imputed datasets provided by NHANES and the resulting parameter estimates were combined with SAS proc mianalyze. Graphs were produced using R version 3.0.1 (The R Foundation for Statistical Computing, Vienna, Austria).

## Results

### Participant characteristics

Mean age of the CKD population was 60.7 (0.7) years with 43.1% males, while 50.6% of non-CKD participants were males with a mean age of 43.9 (0.3) years. A higher proportion of the CKD population had diabetes (20.4% vs. 4.6%), hypertension (62.4% vs. 52.8%), and hyperlipidemia (67.9% vs. 52.8%) in comparison to the non-CKD population (all p-values <0.001, Table 1). Participants with CKD (n = 2,153) had higher waist circumference (101.6 cm vs. 96.1 cm, p < 0.001), BMI (29.2 kg/m^2^ vs. 28.1 kg/m^2^, p < 0.001) and fat mass than those without CKD. In addition, a higher proportion of CKD participants were physically inactive (Table 1). CKD participants had a lower household income compared to non-CKD participants, with 26% of CKD participants earning < $20,000 (vs. 15% of non-CKD population) and 14% of participants earning ≥ $75,000 (vs. 26% of non-CKD population) (Table 1).

**Table 1:** Characteristics of CKD and non-CKD participants in NHANES 1999-2004

**Variable**	**CKD**	**Non-CKD**	P-value**
	**(N = 2153)**	**(N = 9433)**	
Age, years (mean ± SE)	60.7 (0.7)	43.9 (0.3)	<0.001
Male gender, % (SE)	43.1 (1.0)	50.6 (0.4)	<0.001
Race, % (SE)			0.004
Non-Hispanic White	71.9 (2.1)	72.4 (1.6)	
Non-Hispanic Black	10.8 (1.2)	9.9 (0.9)	
Mexican American	5.6 (1.0)	7.6 (0.9)	
Other Hispanic	5.8 (1.4)	5.8 (1.1)	
Other	5.8 (1.0)	4.2 (0.4)	
Smoking, % (SE)	18.7 (1.1)	25.6 (0.8)	<0.001
Income, % (SE)			<0.001
Missing	11.0 (1.0)	9.0 (0.6)	
<20,000	26.3 (1.8)	15.0 (0.8)	
20,000-45,000	30.1 (1.4)	26.5 (1.0)	
45,000-75,000	18.4 (1.6)	23.4 (0.8)	
> = 75,000	14.2 (1.5)	26.1 (1.4)	
BMI (mean ± SE)	29.2 (0.3)	28.1 (0.1)	<0.001
Waist circumference (cm) (mean ± SE)	101.1 (0.6)	96.1 (0.3)	<0.001
Total fat kg (mean ± SE)*	30.3 (0.4)	28.1 (0.2)	<0.001
Total lean mass excluding BMC kg (mean ± SE)*	49.1 (0.4)	51.2 (0.2)	<0.001
Percent body fat (mean ± SE)*	36.7 (0.2)	33.8 (0.1)	<0.001
eGFR, ml/min/1.73 m^2^ (mean ± SE)	72.9 (0.9)	96.8 (0.4)	<0.001
UACR (mg/g) (mean ± SE)	154.4 (15.4)	7.1 (0.08)	<0.001
C-reactive protein (mg/dL)	0.59 (0.03)	0.39 (0.009)	<0.001
Hypertension (SBP >140 or DBP >90 mm Hg) or use of antihypertensives	62.4 (1.6)	25.2 (0.8)	<0.001
Diabetes	20.4 (1.4)	4.6 (0.2)	<0.001
Hyperlipidemia	67.9 (1.5)	52.8 (0.8)	<0.001
Liver condition	3.7 (0.5)	3.1 (0.2)	0.07
Cancer,% (SE)	16.1 (1.1)	6.6 (0.3)	<0.001
Physical activity,% (SE)			<0.001
LTPA <450 METS/week	67.0 (1.7)	53.7 (1.1)	
LTPA 450–749.9 METS/week	8.7 (0.8)	9.1 (0.4)	
LTPA ≥750 METS/week	24.3 (1.5)	37.2 (1.0)	
Sodium intake mg (mean ± SE)***	3011 (45)	3549 (30)	<0.001

*Means and standard errors from survey means on each of the 5 imputations analyzed using procmianalyze.

**Rao-Scott Chi-square test for categorical variables and t-test for continuous variables.

***Sodium available for 2035 and 8095 CKD and non CKD participants respectively.

### Associations between anthropometric measures and death

#### CKD participants

**Figure 2:**
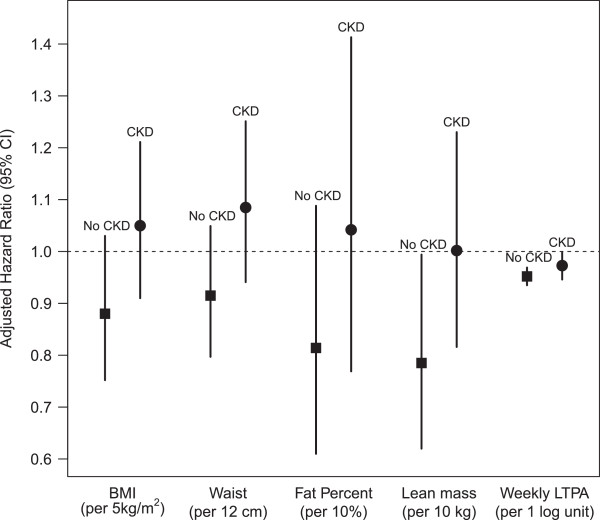
Risk for death with higher BMI, WC, percent body fat, lean body mass and LTPA in CKD and non-CKD participants of the NHANES 1999–2004 survey.

Of 2,153 subjects with CKD included in our study, 363 died. The average person months of follow-up from the time of medical examination was 54.0 (SE = 1.1). Among those with CKD 2,027 participants with 336 deaths had complete covariate data for the mortality models including sodium intake. When adjusting for relevant confounding variables, an increase in BMI, WC, percent body fat or lean body mass were not significantly associated with mortality (Figure 2). The categorical analysis also showed no significant associations between obesity measures (defined by BMI, WC and percent body fat criteria) and mortality (Table 2). We found no significant interaction between any of the anthropometric measures, lean body mass and age, gender, race, diabetes or hypertension.

**Table 2:** Associations between obesity measures (BMI, WC and body fat) and mortality in CKD and non-CKD participants of NHANES 1999-2004

	**CKD**	**Non-CKD**
	Adjusted HR*	Adjusted HR*
	**(95% CI)**	**(95% CI)**
	**N = 2027**	**N = 8881**
**Body mass index**		
25-29.9 kg/m^2^ vs. 18.5-24.9 kg/m^2^	0.84 (0.55, 1.30)	0.62 (0.40, 0.95)
30-34.9 kg/m^2^ vs. 18.5-24.9 kg/m^2^	1.03 (0.71, 1.51)	0.84 (0.56, 1.26)
>35 kg/m^2^ vs. 18.5-24.9 kg/m^2^	1.29 (0.76, 2.19)	0.62 (0.34, 1.15)
**Waist circumference**		
>102 cm in men and >88 cm in women (vs. <102 cm in men and <88 cm in women)	1.04 (0.74, 1.45)	0.87(0.65, 1.16)
Percent body fat**		
>25% in men and >35% in women (vs. <25% in men and <35% in women)	0.87 (0.54, 1.39)	0.70 (0.47, 1.05)

*Adjusted for age, gender, race, smoking, income, log c-reactive protein, cancer, eGFR log albumin-creatinine ratio and sodium intake.

**Hazard ratios are from 5 imputed datasets obtained by survey phreg, and combined by mianalyze.

#### Non-CKD participants

Of 9,433 participants without CKD included in our study, 303 died during follow-up. The average person months of follow-up from the time of medical examination was 57.3 (SE = 1.2). Among those with no CKD 8,881 participants with 288 deaths had complete covariate data for the mortality models including sodium intake. When adjusting for age, gender, race, smoking, house-hold income, c-reactive protein, cancer, eGFR, albumin/creatinine ratio, and sodium intake, an increase in BMI, WC, or percent body fat were not significantly associated with mortality Figure 2). In the categorical analysis, overweight participants had a lower hazard of death than those with BMI 18.5-24.9 kg/m^2^ (Hazard ratio [HR] 0.62, 95% CI 0.40, 0.95) but class I and class II obese participants had no increased risk for death (Table 2). Obesity defined by high WC and high percent body fat criteria were also not significantly associated with mortality (Table 2). On the other hand, each 10 kg increase higher lean body mass was associated with lower mortality (HR 0.78, 95% CI 0.62, 0.99) (Figure 2). We found no significant interaction between any of the anthropometric measures, lean body mass and age, gender, race, diabetes or hypertension.

### Associations between LTPA and mortality

#### CKD

Among those with CKD 2,145 participants with 363 deaths had complete covariate data for the mortality and LTPA models. When adjusting for relevant confounding variables, and percent body fat, LTPA below the recommended level was significantly associated with higher mortality (HR 1.36, 95% CI 1.003, 1.85). However, while adjusting for BMI or waist circumference (instead of percent fat) yielded similar hazard ratios, but these were not statistically significant (Table 3). In the continuous analysis, each log unit increase in METS/week was associated with lower risk for death (HR 0.97, 95% CI 0.95, 1.00) (Figure 2). We found no significant interaction between low levels of LTPA and age, gender, race, diabetes or hypertension.

**Table 3:** Associations of leisure time physical activity (LTPA) with mortality in CKD and non-CKD participants of NHANES 1999-2004^*^

	**CKD**	**Non-CKD**
	Adjusted HR^*^	Adjusted HR^*^
	**(95% CI)**	**(95% CI)**
	**N = 2145**	**N = 9409**
LTPA <450 METS/week vs. ≥450 METS /week (adjusting for Body Mass Index)	1.34 (0.98, 1.84)	1.65 (1.19, 2.28)
LTPA <450 METS/week vs. ≥450 METS /week (adjusting for percent body fat assessed by DEXA)	1.36 (1.003, 1.85)	1.65 (1.21, 2.26)
LTPA <450 METS/week vs. ≥450 METS/week (adjusting for Waist Circumference)	1.33 (0.97, 1.84)	1.64 (1.19, 2.26)

*All models were adjusted for the following variables along with BMI, WC, body fat, percent body fat, or lean body mass as specified above: age, gender, race, smoking, income, log c-reactive protein, cancer, eGFR, log albumin-creatinine ratio; HR-Hazard ratio; CKD-chronic kidney disease; DEXA-dual energy x-ray absorptiometry.

#### Non-CKD

Among those with no CKD 9,409 participants with 302 deaths had complete covariate data for the mortality and LTPA models. When adjusting for relevant confounding variables and body fat, LTPA below the recommended level was significantly associated with higher mortality (Table 3). These results were similar to the model adjusted for demographics, comorbidities, and either measure of adiposity (percent body fat, BMI or waist circumference). We found no significant interaction between LTPA and age, race, diabetes or hypertension. However, we found a significant interaction between low levels of physical activity and gender. LTPA <450 METS/min/week was associated with death in females (HR 2.91, 95% CI 1.60, 5.31) but in males it was not significantly associated with death ( HR 1.25, 95% CI 0.83, 1.89). In the continuous analysis, each log unit increase in METS/week was associated with lower risk for death (HR 0.95, 95% CI 0.93, 0.97) (Figure 2).

## Discussion

In this nationally representative data from the general US population, various measures of adiposity including total and percent body fat were not associated with death in those with and without kidney disease. Higher lean body mass was associated with 18% lower risk for death in those without kidney disease but lean body mass was not associated with death in CKD. Irrespective of kidney function, those who did not meet LTPA goals were at higher risk for death in this cohort. The associations between adiposity measures seem to be similar irrespective of age, gender and race, but gender did modify the associations between LTPA and death in those without CKD (higher risk in females but not in males).

In the general population, pooled analysis of several studies reported that in comparison to normal weight category, being overweight (BMI 25–29.9 kg/m^2^) or with class I obesity (BMI 30.0-34.9 kg/m^2^) were not associated with higher mortality [2]. Overall, our results relating to BMI were similar to previous reports, but we did not find an association between WC and death as reported in earlier studies [23-26]. These differences could be attributed to the shorter follow-up data available in NHANES compared to other reports. Absence of association between percent and total body fat and mortality in the general population may be explained by the differential associations between visceral and subcutaneous adiposity [27,28]. Body fat distribution is one of the major determinants of metabolic health, and visceral adiposity has a stronger correlation with metabolic abnormalities and cardiovascular disease than subcutaneous adipose tissue [27-29]. Visceral fat is metabolically active and is an important site for adipokines such as adiponectin and leptin, that can modulate inflammation and insulin resistance, and impart cardiovascular risk [30].

Even though studies have shown a relationship between obesity and initiation and progression of kidney disease, studies linking obesity (defined as a BMI ≥30 kg/m^2^) and mortality have shown paradoxical results in the CKD population [7,8,31]. Thus the lack of association between higher BMI and mortality is not surprising. BMI, a composite measure of muscle, and subcutaneous and visceral adipose tissue, is widely used to diagnose obesity in clinical practice. Therefore, it has low sensitivity and is likely to give unreliable results in CKD patients who are often old, frail, and lack the muscle mass of healthy individuals. In addition, lower GFR is associated with fluid retention, which further confounds the utility of anthropometric measurement such as BMI in CKD. Data from the REGARDS cohort suggested higher mortality rates for those with higher WC but not with high BMI [9]. However, we did not observe a higher risk for death with higher WC and this may be attributed to the small sample size of the NHANES sample (2153 vs. 5805 in the REGARDS cohort). Preliminary data also indicate that visceral fat may be linked to coronary artery vascular calcification in CKD, highlighting the need for future studies assessing visceral fat in the CKD population [32,33].

Higher lean body mass, a reflection of higher muscle mass, was associated with lower mortality. Few studies have reported this finding in other cohorts without kidney disease and our findings emphasize the importance of preserving muscle mass in the aging population [34]. Higher physical activity is associated with higher lean body mass, and this may be negatively associated with cardiovascular risk factors including diabetes and hypertension, which could explain the lower risk for death. Even though studies using urinary creatinine measurements (a proxy for muscle mass) reported higher risk for death in diabetic nephropathy patients with lower muscle mass, we did not find such associations with lean body mass in the CKD population [35]. Future studies should examine the associations between muscle mass assessed using MRI or CT, with death, in those with and without kidney disease.

Physical activity improves insulin sensitivity and endothelial function and lowers inflammatory markers thereby rendering cardiovascular benefits [36,37]. Higher levels of physical activity are linked to an overall beneficial impact on cardiovascular disease and all-cause mortality in the general population [10-12,38,39]. Greater physical activity is associated with lower albuminuria in nondiabetic women and higher levels of physical activity are associated with a lower risk of decline in kidney function among adults >65 years after accounting for other comorbid conditions [40]. In the NHANES III cohort, physical inactivity was associated with increased mortality in CKD and non-CKD populations [14,15]. Cumulatively, the data suggests that physical activity level, a surrogate measure of their physical fitness, may adversely affect outcomes in those with and without kidney disease. In addition, there are recent reports that longer sitting time is associated with cardiovascular disease and death in the general population, however the lack of consistent data collection in NHANES over the years precluded further analyses [41].

Studies enrolling diabetics and heart failure patients have reported that both fitness and fatness influence cardiovascular risk factors [42,43]. A higher physical fitness levels among those with a higher BMI is associated with a lower prevalence of cardiovascular risk factors and mortality, compared to those with normal BMI and lower fitness level [43-46]. This may also partly explain the obesity paradox noted in heart failure and diabetic patients. It is unclear if fitness and fatness are differentially associated with the individual cardiovascular risk factors and outcomes in CKD. Physical fitness (based on VO_2max_ measures) was assessed in only a limited number of NHANES participants precluding further sensitivity analysis to address this issue.

Strengths of this analysis include the availability of a nationally representative data sample with adequate representation of various ethnic groups in the United States, and availability of longitudinal data to study mortality. In addition, the use of DEXA to directly determine body composition provides a measure that has been validated and correlates highly with other reference methods [47]. However, this analysis is subject to limitations that include the use of a single UACR measurement in the NHANES surveys, which may lead to misclassification of CKD, especially among participants with early-stage CKD. Understanding the limitations, we used eGFR from single serum creatinine measurement. Further, the number of participants with advanced CKD is relatively small (Additional file 1: Table S1) and whether these associations exist among those with advanced kidney disease needs to be confirmed in future studies. In addition, availability of long-term follow-up data might uncover associations that may have been missed in the current study, as some of the ill-effects of adipose tissue take many years to manifest. In addition, we did not obtain visceral adiposity data, which would have helped distinguish the harmful effects of visceral adiposity over subcutaneous adiposity. When we adjusted for diabetes and hypertension, the results remained similar for the non-CKD population, but lost statistical significance for those with CKD (HR 1.32, 95% 0.99, 1.76). These factors were not adjusted for in the multivariate models as lie in the causal pathway between obesity, low physical activity and death in CKD population [48].

## Conclusion

In summary, physical activity, but not adipose tissue measures, is associated with mortality in those with and without CKD in the US population as represented by NHANES. Higher lean body mass, a surrogate of higher muscle mass, is associated with lower risk for death in those without kidney disease. These data highlight the need to find ways to improve physical activity to positively impact health outcomes in those with and without kidney disease.

## Competing interests

SDN is supported by a career development award from the National Center for Research Resources and the National Center for Advancing Translational Sciences, National Institutes of Health (Grant #TR000440). JPK is supported by the National Institutes of Health - RO1 DK089547-01. JDS is supported by NIH/NIDDK (R01 DK085185-01A1) and NIH/NIMH (P60MD00265). Its contents are solely the responsibility of the authors and do not necessarily represent the official view of NCRR or NIH. Information on Re-engineering the Clinical Research Enterprise can be obtained from http://nihroadmap.nih.gov/clinicalresearch/overview. The authors have no relevant financial interest in the study.

## Authors’ contributions

Concept and design of the study: SDN, SA, JDS. Data analysis: SDN, JDS, SA. Interpretation of data and critical revision for intellectual content: SDN, JPK, JDS, SA, Writing the final manuscript, final approval of version to be published: SDN, JPK, JDS, SA. SDN and SA had full access to all of the data in the study and takes responsibility for the integrity of the data and the accuracy of the data analysis. All authors read and approved the final manuscript.

## Presentations

An Abstract describing this study results was presented as an oral abstract presentation during the American Society of Nephrology annual meeting held on Nov 9 2013 in Atlanta, GA.

## Pre-publication history

The pre-publication history for this paper can be accessed here:

http://www.biomedcentral.com/1471-2369/15/108/prepub

## Supplementary Material

Additional file 1: Table S1 Characteristics of CKD participants in NHANES 1999–2004 based on CKD stage. Click here for file
