# Supplementary material for: Adiposity measures, lean body mass, physical activity and mortality: NHANES 1999–2004
Source: BMC Nephrol. 2014 Jul 8;15:108. doi: 10.1186/1471-2369-15-108 (PMC4099406; doi:10.1186/1471-2369-15-108)
Supplement: Additional file 1: Table S1 — Characteristics of CKD participants in NHANES 1999–2004 based on CKD stage. [file 1471-2369-15-108-S1.doc]

**Additional file 1: Table S1.** Characteristics of CKD participants in NHANES 1999-2004 based on CKD stage

| **Variable** | **Stage 1-2**  **N=1073** | **Stage 3**  **N=1008** | **Stage 4**  **N=72** |
| --- | --- | --- | --- |
| Age, years (mean + SE) | 51.1+0.7 | 71.8+0.6 | 73.2+1.5 |
| Male gender, % (SE) | 46.5+1.8 | 38.5+1.4 | 49.6+7.2 |
| Race, % (SE) |  |  |  |
| Non-Hispanic White | 62.1+2.5 | 84.1+1.6 | 70.3+5.4 |
| Non-Hispanic Black | 13.7+1.7 | 7.1+0.9 | 13.3+3.7 |
| Mexican American | 9.1+1.5 | 1.5+0.4 | 2.7+1.3 |
| Other Hispanic | 7.9+1.7 | 3.4+1.5 | 1.7+1.7 |
| Other | 7.2+1.2 | 3.9+0.9 | 11.9+5.1 |
| Smoking, % (SE) | 27.2+1.7 | 8.6+1.5 | 11.5+4.9 |
| Income, % (SE) |  |  |  |
| Missing | 11.8+1.4 | 10.6+1.3 | 1.7+1.0 |
| <20,000 | 23.8+2.3 | 29.6+2.0 | 22.2+6.0 |
| 20,000-45,000 | 27.8+1.8 | 32.0+2.1 | 45.5+7.5 |
| 45,000-75,000 | 19.3+2.0 | 17.0+2.0 | 21.1+6.0 |
| >=75,000 | 17.3+2.4 | 10.8+1.5 | 9.5+3.6 |
| BMI (mean + SE) | 29.7+0.4 | 28.5+0.3 | 30.1+1.0 |
| Waist circumference (cm) (mean + SE) | 101.7+0.9 | 100.1+0.7 | 105.6+2.5 |
| Total fat kg (mean + SE)* | 30.6+0.6 | 29.8+0.5 | 32.0+1.6 |
| Total lean mass excluding BMC kg (mean + SE)* | 51.3+0.5 | 46.4+0.4 | 49.2+2.0 |
| Percent body fat (mean + SE)* | 35.7+0.3 | 37.7+0.3 | 37.9+1.1 |
| eGFR, ml/min/1.73m^2^ (mean + SE) | 93.7+0.7 | 50.2+0.3 | 24.6+0.6 |
| UACR (mg/g) (mean + SE) | 163.3+13.4 | 99.0+19.1 | 929.7+209.1 |
| C-reactive protein (mg/dL) | 0.6+0.1 | 0.5+0.03 | 0.6+0.1 |
| Hypertension (SBP >140 or DBP >90 mm Hg) or use of antihypertensives | 51.4+2.5 | 74.6+1.6 | 80.7+6.2 |
| Diabetes | 21.6+1.7 | 17.8+2.0 | 39.4+7.3 |
| Hyperlipidemia | 61.7+2.3 | 75.0+1.6 | 75.6+5.6 |
| Liver condition | 3.5+0.6 | 3.9+0.9 | 4.7+2.8 |
| Cancer, % (SE) | 9.8+1.33 | 23.3+1.5 | 25.5+5.8 |
| Physical activity, % (SE) |  |  |  |
| LTPA <450 METS/week | 63.0+2.3 | 70.8+1.9 | 84.9+3.7 |
| LTPA 450-749.9 METS/week | 9.3+1.2 | 8.1+1.1 | 6.0+2.9 |
| LTPA >750 METS/week | 27.7+2.1 | 21.0+1.7 | 9.1+3.3 |
| Sodium intake mg (mean + SE)** | 3316+73 | 2679+60 | 2520+189 |

*Means and standard errors from survey means on each of the 5 imputations analyzed using procmianalyze

*Sodium data available for 1015, 954 and 66 participants from stages 1-2, 3 and 4 respectively
